# The Effects of Dietary Pterostilbene on the Immune Response, Antioxidant Function, and Jejunal Structure of Broilers

**DOI:** 10.3390/ani14131851

**Published:** 2024-06-22

**Authors:** Zesheng Yin, Xue Sun, Xuehong Chai, Xin Zhou, Yingjie Wang, Mengru Liu, Xingjun Feng

**Affiliations:** 1 College of Animal Science and Technology, Northeast Agricultural University, Harbin 150030, China; 17645165172@163.com (Z.Y.); 18800434060@163.com (X.S.); cxh990906@163.com (X.C.); 18800430747@163.com (X.Z.); liumengruu@163.com (M.L.); 2 College of Food Science and Engineering, Tianjin University of Science & Technology, Tianjin 300222, China; yjwang@tust.edu.cn

**Keywords:** pterostilbene, plant polyphenol, broiler, antioxidant, jejunal structure, immune function

## Abstract

**Simple Summary:**

As a resveratrol analog, pterostilbene (PTE) exhibits functions including antioxidant and anti-inflammatory. The main objective of this work was to elucidate whether pterostilbene could enhance the immune function and the morphological integrity of the intestine by suppressing inflammatory factors and certain oxidative stresses, thus enhancing the immune capacity of the organism. This study will help to provide greater insight into the molecular mechanism of dietary PTE involved in improving the intestinal health and immune function of broilers.

**Abstract:**

This experiment was carried out to investigate the effect of pterostilbene (PTE) supplementation in feed on Arbor Acres broilers in terms of serum biochemical parameters, immune and inflammatory responses, antioxidant status, and intestinal morphological structure. For a duration of 42 days, a total of 480 1-day-old Arbor Acres broilers were randomly divided into four groups. Each group was assigned to receive either the basal diet or the basal diet supplemented with 200, 400, or 600 mg/kg of PTE. Each treatment consisted of eight replicates, with 15 chicks per replicate. In comparison with the control group, three PTE treatments significantly increased the lymphocyte transformation rate in the spleen of broilers. The automated biochemical analysis, enzyme-linked immunosorbent assay, and RT-qPCR analysis kits found that 400 mg/kg of PTE significantly increased the serum levels of complement C3, IL-4, and iNOS; reduced the serum levels of IL-6, TNF-α, and mRNA levels of the genes IL-6, IL-8, TNF-α, NLRP3, and IFN-γ; significantly improved the activities of antioxidant enzymes including CAT, GSH-Px, and T-SOD in the jejunum; and significantly reduced the MDA contents in the serum and jejunum of broilers. Nikon microscope observations and ImagePro Plus 6.0 measure results found that 400 mg/kg of PTE supplementation significantly reduced the relative length and weight of the jejunum and improved the jejunal villi structure, resulting in increased intestinal villi, deepened crypt, and an enhanced ratio of villi height to crypt depth (VH/CD). RT-qPCR and Western blot found that dietary PTE also resulted in increased mRNA levels of the genes Claudin-2, Occludin, ZO-1, and Sirt1, and decreased NF-κB protein levels in the jejunum. The results of this study demonstrated that dietary PTE improved the immune function and intestinal health of broilers by reducing inflammation and increasing the antioxidant capacity of the animals.

## 1. Introduction

The modern large-scale intensive breeding model has made significant contributions to the efficient production and rapid development of the poultry industry [1]. While this production paradigm offers consumers abundant and affordable poultry products, it concurrently poses serious threats to the physiological functions of poultry. These threats include a compromised immune function, oxidative stress, and intestinal damage [2,3]. In the post-antibiotic era, there is an urgent need to develop novel, safe, and effective feed additives to enhance the health of poultry and pathogen resistance. These additives aim to fill the gap in the feed and farming sectors left by the absence of antibiotics.

Plant phenolic compounds have attracted more attention in the development field of feed additives due to their various biological properties. Some phenolic compounds such as resveratrol, hydroxytyrosol, curcumin, quercetin, and several phenolic acids have been reported to inhibit various pathogenic microorganisms in vitro and in vivo [4,5,6,7]. Resveratrol is naturally distributed in many plant species such as grapes, peanuts, pines, and berries, and has a variety of biological activities, including antioxidant [8], anti-inflammatory [9], cardiovascular-protective [10], and anti-aging [11] properties. However, it is noteworthy that resveratrol has poor water solubility. It also undergoes rapid metabolism in the intestine and liver, which makes it poorly bioavailable. This may limit its bioefficiency and application effect in vivo [12]. Pterostilbene (PTE, trans-3,5-dimethoxy-4′-hydroxystilbene), is a natural dimethylated analog of resveratrol and has two dimethoxy [13] on the benzene ring relative to resveratrol. PTE exhibits superior lipophilicity and metabolic stability to resveratrol, contributing to better cellular uptake and bioactivity than its parent compound resveratrol [14]. PTE is abundant in berries such as blueberries and blackberries and has preventive effects on animals against oxidative stress, and antifungal and anti-cellular infection [15]. PTE has been confirmed to have beneficial effects of varying degrees on animals in vivo and in vitro under various stress conditions. It has been reported that PTE attenuated liver injury of early-weaned piglets by suppressing mitochondrial dysfunction and endoplasmic reticulum stress [16]. Chen et al. confirmed that PTE restored the hepatic antioxidant function of the diaquat-challenged broilers [17].

Hsu et al. found that PTE downregulated the gene expression levels of inflammatory factors and leptin by inhibiting the activation of NF-κB in a system of co-culture of 3T3-L1 adipocytes and RAW 264.7 macrophages and reduced the inflammatory response of 3T3-L1 adipocytes induced by tumor necrosis factor α (TNFα) by decreasing the expression of genes COX-2, iNOS, IL-6, and IL-1β [18].

As an important concourse for nutrient digestion and absorption as well as an immune barrier, the intestine undertakes important physiological functions. At the same time, it continuously encounters complex environmental pressures inside the intestinal cavity such as microbes, antigens, and other harmful substances. These factors can injure the intestine’s structure and lead to inflammation and oxidative function. Therefore, maintaining intestinal health is crucial for promoting the efficient performance of animal production [19,20]. Some reports found that PTE and resveratrol supplementation in diet improved the structure and function of the intestinal mucosal barrier in animals [21,22]. Zhang et al. found that dietary PTE and resveratrol effectively reduced the diamine oxidase (DAO) activity and D-lactate content in the damaged intestine of weaning piglets, with PTE having a more significant effect than resveratrol. These data demonstrated that PTE may be considered as a strategy for the prevention and treatment of intestinal injuries in animals [23]. However, the protective mechanisms of PTE in the intestinal barrier still remain unclear. Therefore, this study for the first time systematically investigates the effects of dietary PTE supplementation on the antioxidant function, inflammatory status, and intestinal structure of Arbor Acres broilers. Meanwhile, it is hoped that this study will provide a theoretical basis for the development of PTE as a feed additive.

## 2. Materials and Methods

### 2.1. Experimental Animal Design and Diets

A total of 480 healthy 1-day-old male Arbor Acres broilers with similar weight were randomly divided into 4 groups, each group containing 8 replicates with 15 broilers per replicate (three chicks per cage). The test period was 42 d. The broilers were housed and fed under standard conditions of care at the Acheng Animal Experimental Base of Northeast Agricultural University. The protocols used for the animal experiments were approved by the Science Ethics Committee of Northeast Agricultural University (NEAUEC20230242). The animal experimental procedures were in accordance with the principles and specific guidelines presented by the Ethics and Animal Welfare Commission of Heilongjiang Province, China. The broilers are raised in a cage (80 cm in length, 60 cm in width, and 60 cm in height). According to experimental requirements, PTE was mixed with the feed in an iron drum using a mixer (JBJ-01, Kwong Wing Fuk Trading Company Limited, Jiaxing, Zhejiang, China) for 30 min to ensure the uniform distribution of PTE in the feed. The control group was fed the basal diet, and the groups of PTE_200_ (basal diet with PTE 200 mg/kg), PTE_400_ (basal diet with PTE 400 mg/kg), and PTE_600_ (basal diet with PTE 600 mg/kg) were fed the basal diet supplemented with 200 mg/kg, 400 mg/kg, and 600 mg/kg of PTE, respectively. The diets were formulated according to the NRC (1994) [24] standard for broiler requirements, and the composition and nutritional levels are shown in Table 1. Mash form and water were offered ad libitum during the test period. On day 7 and day 21, all the broilers received vaccinations against Newcastle disease (ND) and infectious bursal disease (IBD), respectively. During the initial week of age, the ambient temperature (34–35 °C) and humidity (65%–70%) were carefully controlled. Thereafter, both the temperature and humidity levels were gradually reduced by 2–3 °C and 5% per week, respectively, until they stabilized at 23–24 °C and 55%–60%. Throughout the rearing period, the light schedule was 24 h on the 1st and 2nd day, followed by 23 h of continuous light per day. The test period was 42 d.

### 2.2. Sample Collection

On the 42nd day of the experiment, the chickens were weighed and one chicken in replicates closest to the average weight of each replicate was selected for sampling after a fast for 12 h. Blood samples of 3–5 mL were drawn from the jugular vein and collected in a vacuum blood collection tube containing heparin sodium. The tubes were centrifuged at 3000× *g* for 15 min at 4 °C, and the extracted serum was then preserved at −80 °C. After blood collection, the selected chickens were euthanized by bleeding. The following tissues were collected: the thymus, the spleen, a bursa, and the jejunum. The thymus, spleen, and bursa were weighed after the fascia and fatty tissue were removed, and the organ index was determined by dividing the weight of the organ by body weight. The jejunal tissue samples were obtained from the middle section of the intestine. After rinsing the intestine with physiological saline to remove the chyme, a portion of the tissue was fixed in a 4% polyoxymethylene phosphate buffer, and another portion was frozen in liquid nitrogen and stored at −80 °C for further analyses.

### 2.3. Determination of Intestinal Tissue Structure

The jejunal samples were fixed in 4% paraformaldehyde for 24 h, followed by rinsing with distilled water to remove excess paraformaldehyde from the surface of the jejunum. The samples were dehydrated in alcohol, infiltrated with xylene, and embedded in paraffin wax to obtain 5 μm thick specimens. After embedding, the samples were deparaffinized, washed with alcohol, and then immersed in distilled water. The sections were stained with hematoxylin and eosin (H&E) after being soaked in 1% hydrochloric acid alcohol for differentiation. Following staining, the sections were rinsed with distilled water, soaked in alcohol for dehydration, immersed in xylene for transparency, and finally sealed with neutral gum. The method was referenced from Yang et al. [25]. Stained samples were used to assess villi height, crypt depth, and the ratio of villi height to crypt depth (CH/CD). Observations and photographs were taken using a Nikon microscope at 40× (Nikon Eclipse Ci-L, Tokyo, Japan). Eight intact villi were selected from each section, VH and CD were measured using ImagePro Plus 6.0, and the average value of each parameter was calculated for the section.

### 2.4. Measurement of Serum Biochemical Inflammatory Indicators and Immunoglobulins

Serum biochemical factors including total protein (TP), albumin, and globulin were measured using an automated biochemical analyzer (Roche Cobus Mira Plus, Berne, Switzerland) with commercial kits (Nanjing Jiancheng Bioengineering Institute, Nanjing, China). Serum levels of cytokines, including TNF-α, IL-1β, IL-4, IL-6, and IL-8, were measured with ELISA Kits (Sinoukbio, Beijing, China) according to the manufacturer’s instructions. The levels of nitric oxide (NO) and the activities of nitric oxide synthase (NOS) were measured using the relevant kits (Nanjing Jiancheng Bioengineering Institute, China). The immunoglobulin A (IgA), immunoglobulin G (IgG), and immunoglobulin M (IgM) concentrations in the serum and jejunum were determined with the relevant ELISA kits (Wuhan Huamei Biotech Co., Ltd., Wuhan, China) using the double antibody sandwich method.

### 2.5. Measurement of Lymphocyte Transformation Rate of Spleen

Spleen samples were collected after weighing and rinsed 3 times with Hanks buffer without calcium and magnesium. To prepare the cell suspension, the sample was ground in a 200-mesh copper mesh and washed with Hanks buffer. The supernatant was discarded, and the cell precipitate was incubated with three times the volume of red blood cell lysate on ice for 15 min. The spleen cells were prepared by centrifugation at 1200 rpm for 10 min, washed twice, resuspended, and adjusted to 1 × 10^7^ cfu/mL with RPMI 1640 medium. Lymphocyte suspension was added to a 96-well plate, and 45 μg/mL of concanavalin was added. RPMI 1640 medium was used as a control, and each sample was repeated 3 times. After being incubated in a CO_2_ incubator for 48 h, the cells were added CCK-8 and incubated for 2 h. The absorbance of the cells at 450 nm was measured using an enzymatic standard (318MC, Shanghai Sanko Instruments Co., Ltd., Shanghai, China). The lymphocyte transformation rate was expressed as a stimulation index (SI): SI = OD of the experimental group/OD of the control group.

### 2.6. Measurement of Antioxidant Status

The antioxidant indexes of the serum and jejunum were determined with UV-VIS Spectrophotometer (UV1100, MAPADA) using the following assay kits from Nanjing Jiancheng Engineering Institute (Nanjing, China): T-AOC kit (catalog number: A015-1-2); superoxide dismutase (SOD) (catalog number: A001-1-2), glutathione peroxidase (GSH-Px) (catalog number: A005-1-2), catalase (CAT) (catalog number: A007-1-1), and malondialdehyde (MDA) (catalog number: A003-1-2). The experiment was conducted based on the research by Jin et al. [26].

### 2.7. RNA Isolation and Quantitative Real-Time PCR (qRT-PCR)

Total RNA was isolated from frozen jejunum tissues using a Trizol reagent (Takara, Tokyo, Japan) according to the manufacturer’s instructions. RNA concentration and purity were determined using a Nanophotometer P-Class (Implen GmbH, Munich, Germany). Samples with an A260/A280 ratio between 1.8 and 2.0 indicated that the RNA purity was good and could be used for the next PCR reactions. Eligible RNA was reversely transcribed into cDNA using the Reverse Transcription System Kit (Takara, Japan). qRT-PCR was performed using SYBR Green (Takara, Japan) in a PCR instrument (ABI 7500). The expression levels of relevant genes were analyzed with reference to the 2^−ΔΔCt^ method using β-actin as a reference gene. mRNA relative expression levels were calculated using this method, and a bar graph was generated to depict the trend of relative expression changes in the target gene. All PCR primers were synthesized by Sangon Biotech (Shanghai, China), and their sequences are listed in Table 2.

### 2.8. Western Blotting

The jejunal tissue was homogenized and lysed with RIPA buffer (Beyotime Biotechnology, Shanghai, China) containing 1 mM PMSF. Tissue lysate supernatant containing total protein was prepared and the protein concentration was measured with a BCA assay kit (Beyotime Biotechnology). Protein extracts were mixed with an equal volume of loading buffer, separated in 12% SDS-PAGE electrophoresis, and transferred to PVDF membranes. After membrane transfer, blocking, and antibody incubation, protein bands on membranes were detected with a gel documentation system (UVItec, Cambridge, UK) using an ECL chemiluminescence kit (Beyotime Biotechnology). The expression levels of the indicated proteins were quantified numerically and normalized to that of GAPDH. All antibodies were purchased from Shenyang Wanlei Biotechnology Co. (Shenyang, China): p-NFκB (catalog number: WL069), NFκB (catalog number: WL01273b), Sirt-1 (catalog number: WL00599), and GAPDH (catalog number: WL01114).

### 2.9. Statistical Analysis

The results were expressed as mean ± standard deviation (mean ± SD) and analyzed using SPSS 25.0 (SPSS Inc., Chicago, IL, USA). Statistical significance was assessed by one-way ANOVA and an LSD test as a post hoc test. In all statistical comparisons, values of *p* < 0.05 were selected as statistically significant, and all graphs with standard deviation bars were produced by GraphPad Prism (version 8.3.0, GraphPad Software, San Diego, CA, USA).

## 3. Results

### 3.1. Effects of Dietary PTE on Immune Indexes of Broilers

The effects of the PTE supplementation in diet on the immunity organ indexes of broilers are shown in Table 3. PTE supplementation in the diet significantly improved the thymus index of the PTE_600_ group compared to the control group (*p* < 0.05). The spleen and bursal indexes were not significantly affected by PTE (*p* > 0.05).

**Figure 1:**
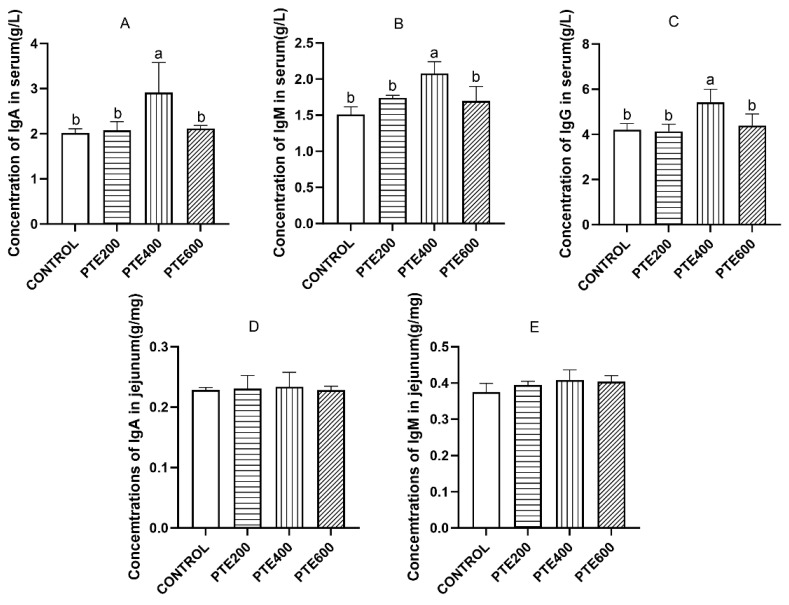
The effects of dietary supplementation with PTE on the levels of immunoglobulins in broilers. (**A**) The level of IgA in serum, (**B**) the level of IgM in serum, (**C**) the level of IgG in serum, (**D**) the level of IgA in the jejunum, and (**E**) the level of IgM in the jejunum. Data are represented as the mean ± SD (*n* = 8), ^a, b^ Values within a row with different letters indicate significant differences (*p* < 0.05) among groups, and the same letters or no letters indicate non-significant differences (*p* > 0.05).

**Figure 2:**
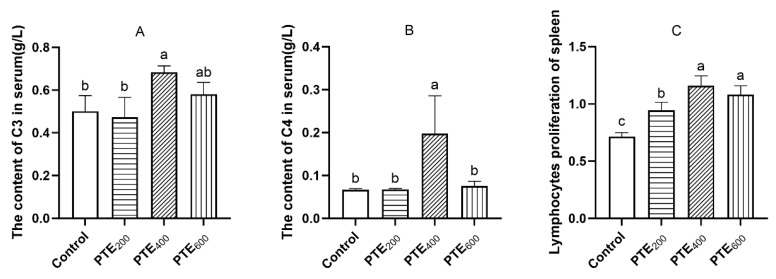
The effects of dietary supplementation with PTE on the lymphocyte proliferation of the spleen and the levels of C3 and C4 in the serum of broilers. (**A**) The level of C3 in serum, (**B**) the level of C4 in serum, and (**C**) the lymphocyte proliferation of the spleen. Data are represented as the mean ± SD (*n* = 8). ^a, b, c^ Values within a row with different letters indicate significant differences (*p* < 0.05) among groups, and the same letters or no letters indicate non-significant differences (*p* > 0.05).

As shown in Figure 1A–C, there were significant differences in the serum levels of IgA, IgM, and IgG among the groups (*p* > 0.05). In the jejunum, there was no statistically significant variation in IgA and IgM contents (*p* > 0.05) (Figure 1D,E). Both serum C3 and C4 levels in the PTE_400_ group were significantly higher than those of the control group (*p* < 0.05) (Figure 2A,B). As shown in Figure 2C, dietary supplementation with PTE significantly influenced the lymphocyte proliferation rate of the spleen (*p* < 0.05), in which the PET_400_ and PET_600_ groups were extremely significantly higher than the control group (*p* < 0.05).

### 3.2. Effects of Dietary PTE on Inflammatory Response of Broilers

As shown in Table 4, compared with the control group, IL-4 levels in the serum of the PTE_200_ and PTE_400_ groups were significantly increased (*p* < 0.05). IL-6 levels in the serum of three PTE groups, IL-1β levels in the serum of the PTE_200_ group, and TNF-α levels in the serum of the PTE_400_ and PTE_600_ groups were significantly decreased compared with the control group (*p* < 0.05). TNOS content in blood was lowered significantly (*p* < 0.05) in the PTE_400_ and PTE_600_ groups, and NO content in blood was dramatically reduced (*p* < 0.05) in the PTE_600_ group compared with those of the control group. The addition of PTE to the diet had no effect on the contents of iNOS in broiler blood compared with the control group (*p* > 0.05).

### 3.3. Effects of Dietary PTE on Antioxidant Status of the Broiler Serum and Jejunum

As Table 5 shows, there were no significant effects of PTE on the serum T-AOC content, CAT activity, and GSH-Px activity compared to the control group (*p* > 0.05). However, compared with the control group, the MDA content in the three PTE groups significantly increased (*p* < 0.05), and the T-SOD activity in the PTE_200_ group significantly increased (*p* < 0.05).

The effects of dietary PTE on the antioxidant status of the broiler jejunum were as shown in Table 6. There was no significant change in T-AOC content in the jejunum among the groups (*p* > 0.05). The three PTE groups had significantly higher CAT activity than the control group (*p* < 0.05). Significant increases in GSH-Px activity in the PTE_400_ (*p* < 0.05) and PTE_600_ groups (p < 0.05) and T-SOD activity in the PTE_400_ group (*p* < 0.05) were found compared with those of the control group. Compared with the control group, the content of MDA in the three PTE treatment groups was significantly decreased (*p* < 0.05).

### 3.4. Effects of Dietary PTE on Intestinal Structure of Broilers

#### 3.4.1. The Jejunum Index

The effects of dietary PTE on the relative length, relative weight, and unit weight of the jejunum are shown in Table 7. Compared to the control groups, the relative length of jejunum in the PTE_400_ group was significantly reduced (*p* < 0.05). Also, the relative weights of jejunum in the PTE_400_ and PTE_600_ groups were significantly decreased (*p* < 0.05). The unit weight of the jejunum in the PTE_600_ group was significantly decreased compared with the other groups (*p* < 0.05).

#### 3.4.2. The Jejunal Structure

**Figure 3:**
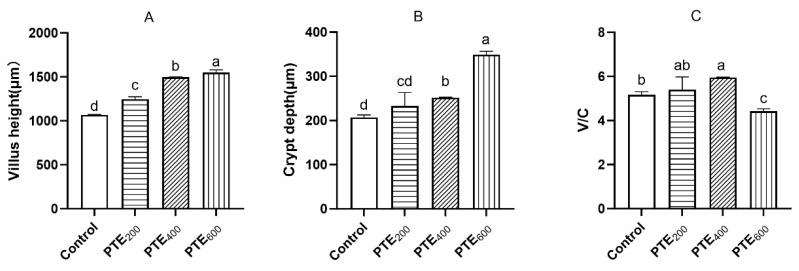
The effects of dietary supplementation with PTE on the jejunum structure of broilers. (**A**) The villus height, (**B**) the crypt depth, and (**C**) the ratio of villus height/the crypt depth. Data are represented as the mean ± SD (*n* = 8). ^a, b, c, d^ Values within a row with different letters indicate significant differences (*p* < 0.05) among groups, and the same letters or no letters indicate non-significant differences (*p* > 0.05).

As shown in Figure 3, the three PTE groups significantly increased the villus height of the jejunum (*p* < 0.05), and both the PTE_400_ and PTE_600_ groups significantly increased the crypt depth compared with the control group (*p* < 0.05). In comparison with the control group, the value of VH/CD in the PTE_400_ group was significantly increased (*p* < 0.05), while the value of VH/CD in the PTE_600_ group was significantly decreased (*p* < 0.05).

### 3.5. The Effects of Dietary PTE on the Expression Levels of Tight Junction Protein Genes in the Jejunum

**Figure 4:**
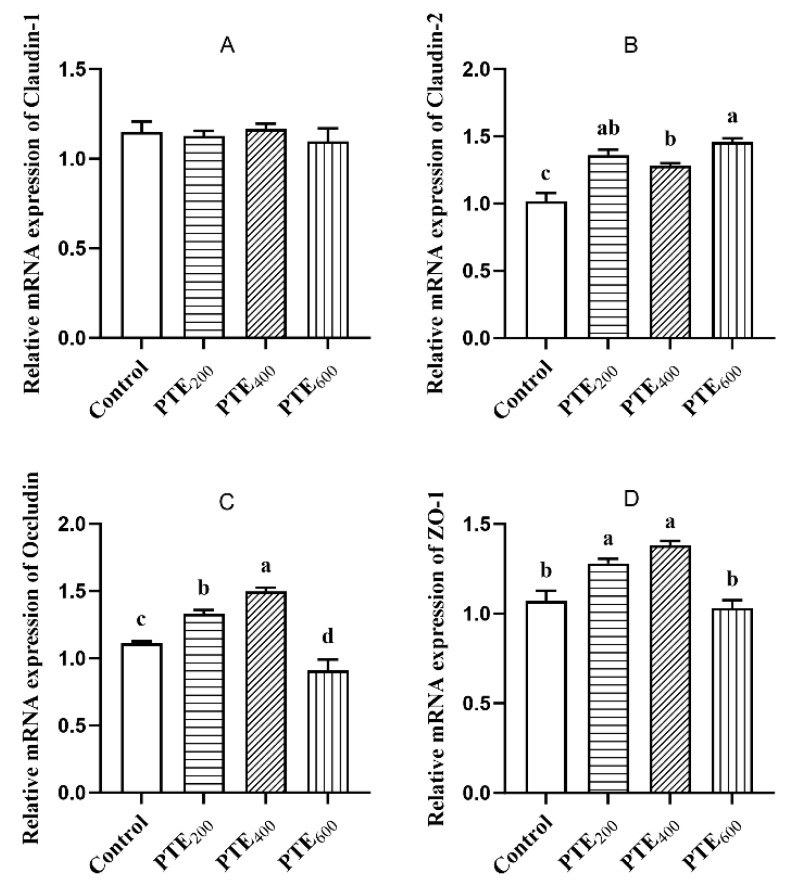
The effects of dietary supplementation with PTE on the expression levels of the tight junction protein genes in the jejunum. (**A**) Claudin-1 mRNA level; (**B**) Claudin-2 mRNA level; (**C**) Occludin mRNA level; (**D**) ZO-1 mRNA level. Data are represented as the mean ± SD (*n* = 8). ^a, b, c, d^ Values within a row with different letters indicate significant differences (*p* < 0.05) among groups, and the same letters or no letters indicate non-significant differences (*p* > 0.05).

As shown in Figure 4, compared with the control group, PTE supplementation in the diet had no significant effect on the relative mRNA expression of Claudin-1 in the jejunum (*p* > 0.05). The Claudin-2 mRNA levels in the three PTE groups were significantly higher than that in the control group, with the PTE_600_ group showing a very significant increase in Claudin-2 mRNA level (*p* < 0.01). The mRNA levels of Occludin and ZO-1 in the PTE_200_ and PTE_400_ groups were significantly increased (*p* < 0.05), but unexpectedly, the mRNA levels of Occludin and ZO-1 in the PTE_600_ group were significantly decreased (*p* < 0.05).

### 3.6. The Effects of Dietary PTE on the Expression Levels of Inflammation-Related Genes in the Jejunum

**Figure 5:**
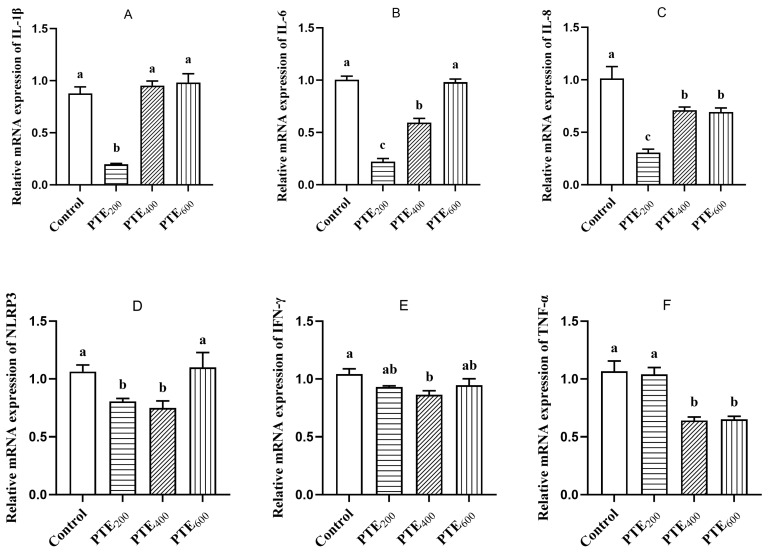
The effects of dietary supplementation with PTE on the mRNA levels of genes related to inflammation in the jejunum. (**A**) IL-1β mRNA level. (**B**) IL-6 mRNA level. (**C**) IL-8 mRNA level. (**D**) NLRP3 mRNA level. (**E**) IFN-γ mRNA level. (**F**) TNF-α mRNA level. Data are represented as the mean ± SD (*n* = 8). ^a, b, c^ Values within a row with different letters indicate significant differences (*p* < 0.05) among groups, and the same letters or no letters indicate non-significant differences (*p* > 0.05).

The effect of dietary supplementation with PTE on the expression of inflammation-related genes in the jejunum of broilers is shown in Figure 5. Compared with the control group, the relative mRNA level of IL-1β in the jejunum of the PTE_200_ group was significantly reduced (*p* < 0.05), while there was no significant change in the relative mRNA levels of IL-1β in the jejunum of the PTE_400_ and PTE_600_ groups (*p* > 0.05). The relative mRNA level of IL-6 in the PTE_400_ group was significantly reduced (*p* < 0.05), with the PTE_200_ group showing a very significant reduction in the relative mRNA level (*p* < 0.01) and no significant change in the PTE_600_ group (*p* > 0.05). In comparison with the control group, the relative mRNA expression of IL-8 was significantly decreased in the three treatment groups of PTE (*p* < 0.05), with a very significant decrease in t in the PTE_200_ group (*p* < 0.01). Compared with the control group, the relative mRNA level of NLRP3 was significantly decreased in the PTE_200_ group (*p* < 0.05), and in the PTE_400_ group, the relative mRNA levels of NLRP3, TNF-α, and IFN-γ were significantly reduced (*p* < 0.05). Additionally, in the PTE_600_ group, the relative levels of TNF-α mRNA were significantly lower (*p* < 0.05).

### 3.7. The Effects of Dietary PTE on the Expression Levels of Sirt1 and NF-κB Genes in the Jejunum

**Figure 6:**
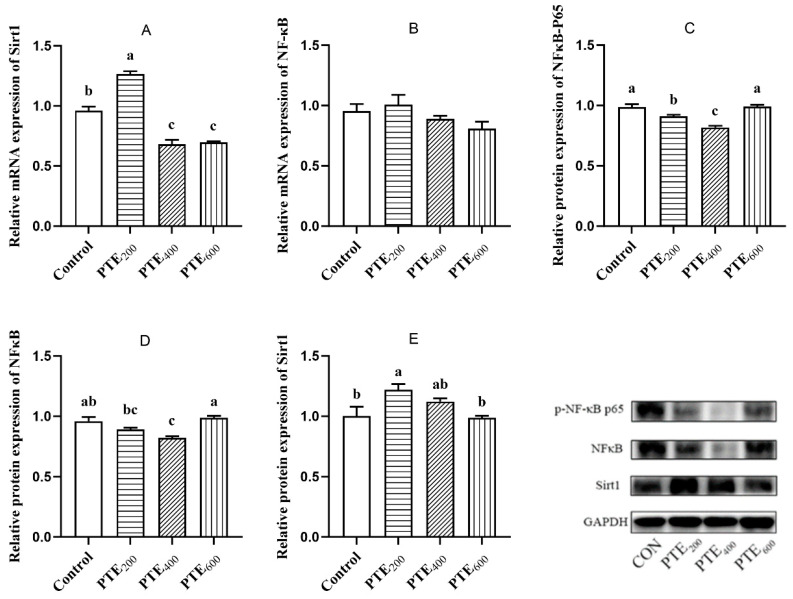
The effects of PTE on the expression levels of Sirt1 and NF-κB protein in the jejunal mucosa of broilers. (**A**) Sirt1 mRNA level. (**B**) NF-κB mRNA level. (**C**) NF-κB protein level. (**D**) p-NF-κB p65 protein level. (**E**) Sirt1 protein level. Data are represented as the mean ± SD (*n* = 8). ^a, b, c^ Values within a row with different letters indicate significant differences (*p* < 0.05) among groups, and the same letters or no letters indicate non-significant differences (*p* > 0.05).

The effects of dietary supplementation with PTE on the expression levels of Sirt1 and NF-κB genes in the jejunum of broilers are shown in Figure 6. Compared with the control group, the mRNA level of Sirt1 in the jejunum was significantly increased in the PTE_200_ group (*p* < 0.05), while it was significantly reduced in the PTE_400_ and PTE_600_ groups (*p* < 0.05). The mRNA level of NF-κB did not show significant changes among the four groups (*p* > 0.05). In comparison with the control group, the level of NF-κB protein in the PTE_400_ group in the jejunum was significantly reduced (*p* < 0.05). The level of p-NF-κB P65 protein in the PTE_200_ and PTE_400_ groups was significantly reduced (*p* < 0.05), with the PTE_600_ group showing a very significant decrease (*p* < 0.01). The protein level of Sirt1 in the PTE_200_ group was significantly increased compared with that of the control group (*p* < 0.05).

## 4. Discussion

Previous studies have demonstrated the attractive preventive and therapeutic properties of PTE in a range of inflammation-related diseases [27,28,29,30]. Our previous research showed that compared with the control group, PTE supplementation increased final body weight (FBW), with significant improvements observed in the PTE_200_ and PTE_400_ groups, the PTE_200_, PTE_400_, and PTE_600_ groups showed significant increases in average daily gain (ADG) [31]. However, the role of PTE in improving organism immunity and intestinal health in broilers remains unclear. Therefore, in this study, we systematically investigated the effects of PTE supplementation in the diet on the antioxidant function, inflammatory status, and intestinal structure of broilers.

The bursa of Fabricius, thymus, and spleen are important immune organs involved in cellular and humoral immunity in poultry [32]. The thymus and bursa of Fabricius are central immune organs [33] and the spleen is an important peripheral immune organ [34]; the growth and development of these immune organs are the basis of immune system function in poultry. The addition of resveratrol to the diet has been reported to improve the indexes of the thymus, spleen, and bursa of Fabricius under heat stress [35,36]. A basal diet with 500 mg/kg resveratrol promoted the proliferation of chicken splenocytes, bursal cells, and thymocytes, and reduced apoptosis. Our results showed a significant increase in the thymus index and an increasing trend in the spleen index and bursa index in broilers of the PTE_600_ group. These results demonstrated that dietary PTE could promote the development and maturation of immune organs, and subsequently improve the immune capacity of broilers.

Immunoglobulins and complements in blood are commonly used to assess the immune status of animals due to their important role in immune function. IgA is an important component of the adaptive humoral immune system and an important component of primary immunoglobulins neutralizing external mucosal surface pathogens while resisting proteases [37,38,39]. IgM is the B-cell antigen receptor and occurs as the first antibody appearing when initial exposure to the neoantigen. IgG is the primary systemic antibody of avians that occurs and acts on infection as a secondary antibody after IgM production [5]. A previous study on the immune function of piglets fed different concentrations of resveratrol (100, 300, and 1000 mg/kg) for 2 weeks showed a significant increase in antibody titers, which suggested that resveratrol could be considered as a feed additive to enhance humoral and cellular immunity [40]. Similar increases in IgG and IgM concentrations after feeding with resveratrol (800 mg/kg) have been found in pigs and broilers [41]. Resveratrol has been reported to have phagocytosis of macrophages and proliferation of lymphocytes in immunosuppressed mice by inhibiting inflammation [42]. In this study, the addition of PTE to feed showed a trend of increasing immunoglobulin levels in the blood and jejunum of broilers, and different doses of PTE in the diet significantly raised the proliferation rate of broiler spleen lymphocytes.

Complement C3 is the most abundant and important component of the complement system and is the central link between the two major activation pathways of complements. It indicates that the total complement level in the serum is an important indicator of humoral immunity. C4 plays an important role in the activation phase of the classical pathway [43], and its stability is very crucial for the maintenance of physical health. It has been demonstrated that heat stress can suppress immune function by decreasing serum C3 and C4 levels, and that recipes supplemented with resveratrol prevented the decrease of serum C3 and C4 levels caused by heat stress [44]. In the present study, the levels of both C3 and C4 in serum were upregulated, with C3 and C4 significantly increased in the PTE_400_ group.

The oxidative metabolite of NOS and NO can inhibit the activity of antioxidant enzymes such as glutathione peroxidase, and decrease the levels of ascorbic acid, uric acid, plasma thiol, and some other cellular antioxidants [45]. It has been shown that NO has distinct roles in organisms. It is an important host defense effector in the immune system, and on the other hand, it is a free oxygen that can act as a cytotoxic agent in pathological processes, especially in inflammatory diseases. The inhibition of iNOS may help in the treatment of inflammatory diseases [46,47,48]. In this study, TNOS levels were significantly lower in the PTE_400_ group and NO content was significantly reduced in the PTE_600_ group compared with those in the control group.

Resveratrol has been reported to show a strong inhibiting effect on alloantigen-induced proliferation of splenic lymphocytes, transformation activity, and NO production in macrophages [49,50]. It also inhibited the induction of nitric oxide synthase and disrupted the metabolism of arachidonic acid by inhibiting cyclooxygenase 2 [51,52]. The results of this study demonstrated that the addition of PTE to the diet increased CAT, T-AOC, T-SOD, and GSH-Px activities in the blood, decreased the MDA content in the jejunum and blood, and enhanced the antioxidant capacity of the organism.

The crypt depth reflects the production rate of the villi epithelium cell, where cells continuously migrate from the base of the crypt to the tip of the villi and subsequently differentiate to form intestinal villi cells with absorptive functions to compensate for the loss of the villi epithelium. The crypt ratio is an indicator of the possible digestibility of the intestine, and an increase in this ratio corresponds to an increase in digestion and absorption [53]. Liu et al. and Zhang et al. reported that broilers fed diets containing 300 mg/kg and 600 mg/kg resveratrol showed improved VH/CD ratios in the duodenum and jejunum [54,55]. Broilers fed 60 g/kg of grape seed extract containing high amounts of polyphenols including resveratrol also showed longer villi length and improved intestinal health. Consistent with these previous findings, the results of this study demonstrated that PTE increased the VH, CD, and the VH/CD ratio in the jejunum, which indicates improvement in the morphology integrity and function of the intestine.

Healthy gut function is crucial for the growth performance and well-being of animals. Previous studies have shown that resveratrol supplementation can improve intestinal integrity and barrier function by stimulating the growth of beneficial bacteria, inhibiting pathogen colonization, and modulating the immune system [41,56]. The structure barrier integrity of the intestine is usually regulated by tight junctional complexes between epithelial cells. Several studies have shown that resveratrol and its analogs can modulate the barrier function of the gastrointestinal tract [57,58]. Bereswill et al. and Ling et al. evaluated the protective effects of resveratrol in a mouse model of acute intestinal inflammation and showed that oral resveratrol prevented bacterial translocation by maintaining intestinal integrity [59,60]. Similarly, an in vitro study by Ling et al. showed that pretreatment of cell monolayers with resveratrol preserved intestinal barrier function by strengthening the physical barrier [60]. Polydatin, a glucoside of resveratrol, was also found to attenuate oxidative damage and epithelial barrier disruption in rat colon [57]. Increased expression of Claudin-2, Occlduin, and ZO-1 was seen in the jejunum of broilers treated with PTE in this study, which demonstrated that PTE as a protective agent can prevent intestinal mucosal damage and preserve the morphological structure of the jejunum in broilers.

Since a well-regulated inflammatory process is essential for homeostasis in the intestine, an uncontrolled and excessive inflammatory response can cause damage to the intestine. NF-κB is a widely expressed transcription factor that primarily regulates immune and inflammatory responses. Sirt1 has been proven to be a key signal factor regulating intestinal barrier function [61,62]. Sirt1 activation effectively alleviated colitis in mice induced by dextran sulfate sodium [63]. In the present study, a decrease in nuclear NF-κb p65 protein and a decrease in the release of pro-inflammatory cytokines, such as IL-1β, IL-6, IL-8, TNF-α, IFN-γ, NF-κB, and NLRP3 expression in broiler jejunum were observed compared with those of the control group, which may ameliorate intestinal inflammation and concomitant oxidative damage.

## 5. Conclusions

In summary, PTE supplementation in the diet resulted in improved immune and antioxidant functions, and improved intestinal health of broilers, which mainly depended on the antioxidant and anti-inflammatory properties of PTE. Administration of PTE improved immune organ indexes, increased the serum level of the C3 and C4 complement and the secretion of anti-inflammatory factors including IL-4, and reduced the level of pro-inflammatory factors including IL-1β, IL-6, IL-8, TNF-α, IFN-γ, NLRP3, TNOS, and NO. PTE significantly increased the activity of CAT, GSH-Px in the jejunum, and T-SOD in the jejunum and blood, and decreased MDA content in the jejunum and blood, which strongly suggested that dietary PTE effectively increased the antioxidant capacity of broilers. Dietary PTE improved the structure and health of the intestine by increasing the expression of tight junction protein genes including Claudin-1, Claudin-1, Occludin, and ZO-1. PTE regulation of inflammation was mainly by decreasing the expression of NF-κb, increasing the expression of Sirt-1, and upregulating the expression of downstream antioxidant enzymes. Therefore, PTE could enhance the immune function and the morphological integrity of the intestine by suppressing inflammatory factors and certain oxidative stresses, thus enhancing the immune capacity of the organism. This study will help to provide greater insight into the molecular mechanism of dietary PTE involved in improving the intestinal health and immune function of broilers.

## Figures and Tables

**Table 1:** Composition and nutrition levels of the experimental basal diets.

Ingredients (%)	0–21 Days	22–42 Days	Nutrient Levels ^2^	0–21 Days	22–42 Days
Corn	58.50	61.15	Metabolic energy (MJ/kg)	12.54	12.96
Soybean meal	30.00	26.30	Crude protein (%)	21.50	20.09
Soybean oil	2.70	3.80	Lysine (%)	1.15	1.01
Corn gluten meal	4.06	4.33	Methionine (%)	0.55	0.43
Methionine	0.21	0.10	Methionine + Cysteine (%)	0.91	0.77
L-Lysine	0.20	0.14	Threonine (%)	0.80	0.73
Dicalcium phosphate	1.60	1.52	Tryptophan (%)	0.21	0.18
Limestone	1.33	1.26	Arginine (%)	1.20	1.12
Sodium chloride	0.30	0.30	Leucine (%)	1.26	1.05
Choline chloride	0.10	0.10	Isoleucine (%)	0.81	0.75
Premix ^1^	1.00	1.00	Phenylalanine (%)	0.71	0.66
Total	100.00	100.00	Phenylalanine + Tyrosine (%)	1.27	1.15
			Histidine (%)	0.35	0.32
			Valine (%)	0.85	0.74
			Calcium (%)	1.06	0.91
			Total phosphorus (%)	0.73	0.69
			Available phosphorus (%)	0.45	0.43

^1^ Provided the following per kilogram of diet: vitamin A, 12,000 IU; vitamin D3, 2500 IU; vitamin E, 20 IU; vitamin K3, 1.3 mg; thiamine, 2.2 mg; riboflavin, 8.0 mg; nicotinamide, 40 mg; calcium pantothenate, 10 mg; pyridoxine, 4 mg; biotin, 0.04 mg; folic acid, 1 mg; vitamin B_12_, 0.013 mg; Fe (from ferrous sulfate), 80 mg; Cu (from copper sulfate), 8.0 mg; Mn (from manganese sulfate), 110 mg; Zn (from zinc sulfate), 60 mg; I (from calcium iodate), 1.1 mg; Se (from sodium selenite), 0.3 mg. ^2^ Data are calculated values.

**Table 2:** Primers for qRT-PCR analysis.

Genes	Sequence (5′ to 3′)	Product Size (bp)	GenBank No.
β-actin	F: TGCGTGACATCAAGGAGAAG R: TGCCAGGGTACATTGTGGTA	300	NM_205518.2
IL-1β	F: TGCCTGCAGAAGAAGCCTCG R: GACGGGCTCAAAAACCTCCT	204	NM_204524.2
IL-4	F: AGCCAGCACTGCCACAAGAAC R: GTGGAAGAAGGTACGTAGGTCTGC	156	XM_046900385.1
IL-6	F: TTTATGGAGAAGACCGTGAGG R: TGTGGCAGATTGGTAACAGAG	106	NM_204628.2
IL-8	F: TCATGTTCTCCATACCCTTGGT R: AAACTGCGAGTGGGGTCAG	175	NM_010851
TNF-α	F: CAGGACAGCCTATGCCAACAAG R: GGTTACAGGAAGGGCAACTCATC	114	XM_046927265.1
IFN-γ	F: ATGTAGCTGACGGTGGACCT R: TTCACGCCATCAGGAAGGTT	196	NM_205149.2
NLRP3	F: GGTTTACCAGGGGAA ATGAG R: TTGTGCTTCCAGAT GCCGT	253	XM_046918112.1
Claudin 1	F: GCAGATCCAGTGCAAGGTGTA R: CACTTCATGCCCGTCACAG	132	NM_001013611.2
Claudin 2	F: CCTGCTCACCCTCATTGGAG R: GCTGAACTCACTCTTGGGCT	145	NM_001277622.1
Occludin	F: CCGTAACCCCGAGTTGGAT R: ATTGAGGCGGTCGTTGATG	214	XM_046904540.1
ZO-1	F: TGTAGCCACAGCAAGAGGTG R: CTGGAATGGCTCCTTGTGGT	98	XM_046925214.1
NF-κB	F: TCAACGCAGGACCTAAAGACAT R: GCAGATAGCCAAGTTCAGGATG	162	XM_046915553.1
Sirt1	F: GATCAGCAAAAGGCTGGATGGT R: ACGAGCCGCTTTCGCTACTAC	143	NM_001004767.2

**Table 3:** The effects of dietary supplementation with PTE on the immunity organ indexes of broilers.

	Control	PTE_200_	PTE_400_	PTE_600_
Thymus index (g/kg·BW)	4.33 ± 0.05 ^a^	4.13 ± 0.14 ^ab^	4.54 ± 0.17 ^a^	3.83 ± 0.15 ^b^
Spleen index (g/kg·BW)	1.35 ± 0.07	1.51 ± 0.010	1.54 ± 0.05	1.29 ± 0.09
Bursa of Fabricius index (g/kg·BW)	1.49 ± 0.04	1.54 ± 0.04	1.63 ± 0.05	1.62 ± 0.09

Each mean ± SD represents eight individuals (*n* = 8). ^a, b^ Values within a row with different superscript letters indicate significant differences (*p* < 0.05) among groups, and the same superscript letters or no superscript letters indicate non-significant differences (*p* > 0.05).

**Table 4:** Effects of dietary supplementation with PTE on inflammatory response of broilers.

Parameters	Control	PTE_200_	PTE_400_	PTE_600_
IL-4 (μg/L)	68.86 ± 1.47 ^b^	77.55 ± 2.50 ^a^	78.37 ± 1.06 ^a^	65.38 ± 2.25 ^b^
IL-6 (μg/L)	76.39 ± 1.94 ^a^	64.91 ± 1.31 ^b^	60.53 ± 0.99 ^b^	65.70 ± 2.48 ^b^
IL-1β (μg/L)	55.1 ± 2.08 ^a^	48.9 ± 1.50 ^b^	52.5 ± 0.911 ^ab^	55.8 ± 1.77 ^a^
TNF-α (pg/mL)	72.9 ± 1.65 ^a^	69.1 ± 1.87 ^a^	63.1 ± 2.40 ^b^	63.4 ± 1.26 ^b^
TNOS (U/mL)	9.87 ± 0.31 ^a^	9.53 ± 0.15 ^ab^	9.19 ± 0.45 ^b^	8.99 ± 0.31 ^b^
iNOS (U/mL)	2.16 ± 0.23	2.51 ± 0.39	2.55 ± 0.37	2.46 ± 0.94
NO (μmol/L)	18.83 ± 1.43 ^ab^	14.54 ± 3.85 ^bc^	19.68 ± 1.70 ^a^	11.23 ± 2.58 ^c^

Data are represented as the mean ± SD (*n* = 8). ^a, b, c^ Values within a row with different superscript letters indicate significant differences (*p* < 0.05) among groups, and the same superscript letters or no superscript letters indicate non-significant differences (*p* > 0.05).

**Table 5:** Effects of dietary supplementation with PTE on serum antioxidant status of broilers.

Items	Control	PTE_200_	PTE_400_	PTE_600_
T-AOC (U/mL)	1.12 ± 0.114	1.53 ± 0.270	1.19 ± 0.187	1.10 ± 0.084
CAT (U/mL)	3.50 ± 0.195	4.44 ± 0.373	4.41 ± 0.744	3.96 ± 0.272
GSH-Px (U/mL)	1001 ± 122	1002 ± 230	1498 ± 126	1378 ± 115
T-SOD (U/mg prot)	127 ± 5.95 ^b^	137 ± 1.14 ^a^	135 ± 0.856 ^ab^	126 ± 4.48 ^ab^
MDA (nmol/mL)	4.27 ± 0.390 ^a^	3.01 ± 0.262 ^b^	3.07 ± 0.183 ^b^	3.41 ± 0.331 ^b^

Data are represented as the mean ± SD (*n* = 8). ^a, b^ Values within a row with different superscript letters indicate significant differences (*p* < 0.05) among groups, and the same superscript letters or no superscript letters indicate non-significant differences (*p* > 0.05). T-AOC = total antioxidant capacity; CAT = catalase; MDA = malondialdehyde; GSH-Px = glutathione peroxidase; T-SOD = superoxide dismutase.

**Table 6:** The effects of dietary supplementation with PTE on the antioxidant status of the jejunum.

Items	Control	PTE_200_	PTE_400_	PTE_600_
T-AOC (U/mg prot)	0.695 ± 0.013	0.699 ± 0.004	0.715 ± 0.008	0.714 ± 0.011
CAT (U/mg prot)	3.824 ± 0.336 ^b^	6.381 ± 0.226 ^a^	7.203 ± 0.176 ^a^	6.876 ± 0.914 ^a^
GSH-Px (U/mg prot)	9.332 ± 1.41 ^c^	12.503 ± 0.916 ^bc^	13.410 ± 0.818 ^b^	22.404 ± 1.190 ^a^
T-SOD (U/mg prot)	102.1 ± 3.1 ^b^	106.4 ± 6.2 ^b^	125.7 ± 3.7 ^a^	105.0 ± 4.0 ^b^
MDA (nmol/mg prot)	0.393 ± 0.0349 ^a^	0.243 ± 0.0137 ^b^	0.0758 ± 0.0324 ^c^	0.389 ± 0.0148 ^a^

Data are represented as the mean ± SD (*n* = 8). ^a, b, c^ Values within a row with different superscript letters indicate significant differences (*p* < 0.05) among groups, and the same superscript letters or no superscript letters indicate non-significant differences (*p* > 0.05).

**Table 7:** The effects of dietary supplementation with PTE on the jejunum index of broilers.

Items	Relative Length (cm/kg·BW)	Relative Weight (g/kg·BW)	Unit Weight (g/cm)
Control	28.98 ± 1.41 ^a^	10.28 ± 0.18 ^a^	0.37 ± 0.02 ^a^
PTE_200_	26.93 ± 1.63 ^ab^	9.92 ± 0.12 ^ab^	0.36 ± 0.01 ^a^
PTE_400_	25.76 ± 0.52 ^b^	9.60 ± 0.07 ^b^	0.36 ± 0.02 ^a^
PTE_600_	27.27 ± 2.57 ^ab^	9.90 ± 0.33 ^b^	0.33 ± 0.15 ^b^

Each mean (±SD) represents six individuals (*n* = 8). ^a, b^ Values within a row with different superscript letters indicate significant differences (*p* < 0.05) among groups, and the same superscript letters or no superscript letters indicate non-significant differences (*p* > 0.05).

## Data Availability

Data are available upon request.

## References

[1] Rashid Z., Mirani Z.A., Zehra S., Gilani S.M.H., Ashraf A., Azhar A., Al-Ghanim K., Al-Misned F., Al-Mulahim N., Mahboob S. (2020). Enhanced modulation of gut microbial dynamics affecting body weight in birds triggered by natural growth promoters administered in conventional feed. SAUDI J. Biol. Sci..

[2] Sejian V., Silpa M., Reshma Nair M., Devaraj C., Krishnan G., Bagath M., Chauhan S., Suganthi R., Fonseca V., König S. (2021). Heat stress and goat welfare: Adaptation and production considerations. Animals.

[3] Chai X., Sun X., Qi X., Shan A., Feng X. (2024). Food Security: Nutritional characteristics, feed utilization status and limiting factors of aged brown rice. Agriculture.

[4] Seigner J., Junker-Samek M., Plaza A., D ‘Urso G., Masullo M., Piacente S., Holper-Schichl Y.M., de Martin R. (2019). A Symphytum officinale root extract exerts anti-inflammatory properties by affecting two distinct steps of NF-κB signaling. Front. Pharmacol..

[5] Moreno R.M., Jimenez V., Monroy F.P. (2019). Impact of Binge Alcohol Intoxication on the Humoral Immune Response during Burkholderia spp. Infections. Microorganisms.

[6] Zhang J., Sun X., Chai X., Jiao Y., Sun J., Wang S., Yu H., Feng X. (2024). Curcumin Mitigates Oxidative Damage in Broiler Liver and Ileum Caused by Aflatoxin B1-Contaminated Feed through Nrf2 Signaling Pathway. Animals.

[7] Guan P., Yu H., Wang S., Sun J., Chai X., Sun X., Qi X., Zhang R., Jiao Y., Li Z. (2024). Dietary rutin alleviated the damage by cold stress on inflammation reaction, tight junction protein and intestinal microbial flora in the mice intestine. J. Nutr. Biochem..

[8] Meng Q., Guo T., Li G., Sun S., He S., Cheng B., Shi B., Shan A. (2018). Dietary resveratrol improves antioxidant status of sows and piglets and regulates antioxidant gene expression in placenta by Keap1-Nrf2 pathway and Sirt1. J. Anim. Sci. Biotechnol..

[9] Nunes S., Danesi F., Del Rio D., Silva P. (2018). Resveratrol and inflammatory bowel disease: The evidence so far. Nutr. Res. Rev..

[10] Dyck G.J., Raj P., Zieroth S., Dyck J.R., Ezekowitz J.A. (2019). The effects of resveratrol in patients with cardiovascular disease and heart failure: A narrative review. Int. J. Mol. Sci..

[11] Liu T.-H., Wang J., Zhang C.-Y., Zhao L., Sheng Y.-Y., Tao G.-S., Xue Y.-Z. (2023). Gut microbial characteristical comparison reveals potential anti-aging function of Dubosiella newyorkensis in mice. Front. Endocrinol..

[12] Wang L., Lai C., Li D., Luo Z., Liu L., Jiang Y., Li L. (2022). Lecithin-polysaccharide self-assembled microspheres for resveratrol delivery. Antioxidants.

[13] Lange K.W., Li S. (2018). Resveratrol, pterostilbene, and dementia. Biofactors.

[14] Wang P., Sang S. (2018). Metabolism and pharmacokinetics of resveratrol and pterostilbene. Biofactors.

[15] Virgili F., Marino M. (2008). Regulation of cellular signals from nutritional molecules: A specific role for phytochemicals, beyond antioxidant activity. Free. Radic. Biol. Med..

[16] Zhang H., Chen Y., Li Y., Jia P., Ji S., Chen Y., Wang T. (2020). Protective effects of pterostilbene against hepatic damage, redox imbalance, mitochondrial dysfunction, and endoplasmic reticulum stress in weanling piglets. J. Anim. Sci..

[17] Chen Y., Chen Y., Zhang H., Wang T. (2020). Pterostilbene as a protective antioxidant attenuates diquat-induced liver injury and oxidative stress in 21-day-old broiler chickens. Poult. Sci..

[18] Hsu C.L., Lin Y.J., Ho C.T., Yen G.C. (2013). The inhibitory effect of pterostilbene on inflammatory responses during the interaction of 3T3-L1 adipocytes and RAW 264.7 macrophages. J. Agric. Food. Chem..

[19] Klingensmith N.J., Fay K.T., Swift D.A., Bazzano J.M., Lyons J.D., Chen C.-W., Meng M., Ramonell K.M., Liang Z., Burd E.M. (2022). Junctional adhesion molecule-A deletion increases phagocytosis and improves survival in a murine model of sepsis. JCI Insight.

[20] Yan D., Wei G., Ai Z., Song S., Zhang L., Dong N., Dou X., Shan A. (2024). CXCR2, as a key regulatory gene of HDP-PG-1, maintains intestinal mucosal homeostasis. J. Agric. Food Chem..

[21] Cai T.-T., Ye X.-L., Li R.-R., Chen H., Wang Y.-Y., Yong H.-J., Pan M.-L., Lu W., Tang Y., Miao H. (2020). Resveratrol modulates the gut microbiota and inflammation to protect against diabetic nephropathy in mice. Front. Pharmacol..

[22] Chen Y., Zhang H., Li Y., Wang T. (2023). Pterostilbene Confers Protection against Diquat-Induced Intestinal damage with potential regulation of redox status and ferroptosis in broiler chickens. Oxid. Med. Cell. Longev..

[23] Zhang H., Chen Y., Chen Y., Ji S., Jia P., Li Y., Wang T. (2020). Comparison of the protective effects of resveratrol and pterostilbene against intestinal damage and redox imbalance in weanling piglets. J. Anim. Sci. Biotechnol..

[24] Council N. (1994). Nutrient Requirements of Poultry.

[25] Yang H., Wang Y., Jin S., Pang Q., Shan A., Feng X. (2022). Dietary resveratrol alleviated lipopolysaccharide-induced ileitis through Nrf2 and NF-κB signalling pathways in ducks (Anas platyrhynchos). J. Anim. Physiol. Anim. Nutr..

[26] Jin S., Yang H., Jiao Y., Pang Q., Wang Y., Wang M., Shan A., Feng X. (2021). Dietary curcumin alleviated acute ileum damage of ducks (Anas platyrhynchos) induced by AFB1 through regulating Nrf2-ARE and NF-κB signaling pathways. Foods.

[27] Choo Q.Y., Yeo S.C.M., Ho P.C., Tanaka Y., Lin H.S. (2014). Pterostilbene surpassed resveratrol for anti-inflammatory application: Potency consideration and pharmacokinetics perspective. J. Funct. Foods.

[28] Zhang L., Zhang L.L., Zhan X.A., Zeng X.F., Zhou L., Cao G.T., Chen A.G., Yang C.M. (2016). Effects of dietary supplementation of probiotic, Clostridium butyricum, on growth performance, immune response, intestinal barrier function, and digestive enzyme activity in broiler chickens challenged with Escherichia coli K88. J. Anim. Sci. Biotechnol..

[29] Kosuru R., Kandula V., Rai U., Prakash S., Xia Z., Singh S. (2018). Pterostilbene Decreases Cardiac Oxidative Stress and Inflammation via Activation of AMPK/Nrf2/HO-1 Pathway in Fructose-Fed Diabetic Rats. Cardiovasc. Drugs Ther..

[30] Chang J., Rimando A., Pallas M., Camins A., Porquet D., Reeves J., Shukitt-Hale B., Smith M.A., Joseph J.A., Casadesus G. (2012). Low-dose pterostilbene, but not resveratrol, is a potent neuromodulator in aging and Alzheimer’s disease. Neurobiol. Aging.

[31] Zhang L., Zhang J., Zang H., Yin Z., Guan P., Yu C., Shan A., Feng X. (2024). Dietary pterostilbene exerts potential protective effects by regulating lipid metabolism and enhancing antioxidant capacity on liver in broilers. J. Anim. Physiol. Anim. Nutr..

[32] Liu L.L., He J.H., Xie H.B., Yang Y.S., Li J.C., Zou Y. (2014). Resveratrol induces antioxidant and heat shock protein mRNA expression in response to heat stress in black-boned chickens. Poult. Sci..

[33] Zhang Y., Zhou Y., Sun G., Li K., Li Z., Su A., Liu X., Li G., Jiang R., Han R. (2018). Transcriptome profile in bursa of Fabricius reveals potential mode for stress-influenced immune function in chicken stress model. BMC Genom..

[34] Xu M., Li W., Yang S., Sun X., Tarique I., Yang P., Chen Q. (2020). Morphological characterization of postembryonic development of blood–spleen barrier in duck. Poult. Sci..

[35] Zhang C., Chen K.K., Zhao X.H., Geng Z.Y. (2018). Protective effects of resveratrol against high ambient temperature-induced spleen dysplasia in broilers through modulating splenic redox status and apoptosis. J. Sci. Food Agric..

[36] He S.P., Yu Q.F., He Y.J., Hu R.Z., Xia S.T., He J.H. (2019). Dietary resveratrol supplementation inhibits heat stress-induced high-activated innate immunity and inflammatory response in spleen of yellow-feather broilers. Poult. Sci..

[37] Berkeveld M., Langendijk P., Verheijden J.H.M., Taverne M.A.M., van Nes A., van Haard P., Koets A.P. (2008). Citrulline and intestinal fatty acid-binding protein: Longitudinal markers of postweaning small intestinal function in pigs?. J. Anim. Sci..

[38] Iizuka M., Konno S. (2011). Wound healing of intestinal epithelial cells. World J. Gastroenterol..

[39] Hermans D., Pasmans F., Heyndrickx M., Van Immerseel F., Martel A., Deun K., Haesebrouck F. (2012). A tolerogenic mucosal immune response leads to persistent Campylobacter jejuni colonization in the chicken gut. Crit. Rev. Microbiol..

[40] Fu Q.T., Cui Q.K., Yang Y., Zhao X.H., Song X., Wang G.X., Bai L., Chen S.F., Tian Y., Zou Y.F. (2018). Effect of Resveratrol Dry Suspension on Immune Function of Piglets. Evid.-Based Complement. Altern. Med..

[41] Viveros A., Chamorro S., Pizarro M., Arija I., Centeno C., Brenes A. (2011). Effects of dietary polyphenol-rich grape products on intestinal microflora and gut morphology in broiler chicks. Poult. Sci..

[42] Lai X., Pei Q., Song X., Zhou X., Yin Z., Jia R., Zou Y., Li L., Yue G., Liang X. (2016). The enhancement of immune function and activation of NF-κB by resveratrol-treatment in immunosuppressive mice. Int. Immunopharmacol..

[43] Wouters D., Wiessenberg H.D., Hart M., Bruins P., Voskuyl A., Daha M.R., Hack C.E. (2005). Complexes between C1q and C3 or C4: Novel and specific markers for classical complement pathway activation. J. Immunol. Methods.

[44] Pasternack M.S. (1994). Fundamental Immunology Edited by William E. Paul. 3rd edition. New York: Raven Press, 1993. 1,490 pp. illustrated. $95. Clin. Infect. Dis..

[45] Han Y.J., Kwon Y.G., Chung H.T., Lee S.K., Simmons R.L., Billiar T.R., Kim Y.M. (2001). Antioxidant enzymes suppress nitric oxide production through the inhibition of NF-kappa B activation: Role of H_2_O_2_ and nitric oxide in inducible nitric oxide synthase expression in macrophages. Nitric Oxide.

[46] Bogdan C. (2001). Nitric oxide and the immune response. Nat. Immunol..

[47] Aktan F., Henness S., Roufogalis B.D., Ammit A.J. (2003). Gypenosides derived from Gynostemma pentaphyllum suppress NO synthesis in murine macrophages by inhibiting iNOS enzymatic activity and attenuating NF-kappaB-mediated iNOS protein expression. Nitric Oxide.

[48] Kroncke K.D. (2001). Cysteine-Zn^2+^ complexes: Unique molecular switches for inducible nitric oxide synthase-derived NO. FASEB J..

[49] Gao X., Xu Y.X., Janakiraman N., Chapman R.A., Gautam S.C. (2001). Immunomodulatory activity of resveratrol: Suppression of lymphocyte proliferation, development of cell-mediated cytotoxicity, and cytokine production. Biochem. Pharmacol..

[50] Radkar V., Hardej D., Lau-Cam C., Billack B. (2007). Evaluation of resveratrol and piceatannol cytotoxicity in macrophages, T cells, and skin cells. Arh. Hig. Rada Toksikol..

[51] Hsieh T.C., Wu J.M. (1999). Differential effects on growth, cell cycle arrest, and induction of apoptosis by resveratrol in human prostate cancer cell lines. Exp. Cell Res..

[52] Schneider Y., Vincent F., Duranton B., Badolo L., Gossé F., Bergmann C., Seiler N., Raul F. (2000). Anti-proliferative effect of resveratrol, a natural component of grapes and wine, on human colonic cancer cells. Cancer Lett..

[53] Montagne L., Pluske J., Hampson D. (2003). A review of interactions between dietary fibre and the intestinal mucosa, and their consequences on digestive health in young non-ruminant animals. Anim. Feed Sci..

[54] Liu L., Fu C., Yan M., Xie H., Li S., Yu Q., He S., He J. (2016). Resveratrol modulates intestinal morphology and HSP70/90, NF-κB and EGF expression in the jejunal mucosa of black-boned chickens on exposure to circular heat stress. Food Funct..

[55] Zhang H., Chen Y., Chen Y., Li Y., Jia P., Ji S., Zhou Y., Wang T. (2020). Dietary pterostilbene supplementation attenuates intestinal damage and immunological stress of broiler chickens challenged with lipopolysaccharide. J. Anim. Sci..

[56] Zhang C., Zhao X., Yang L., Chen X., Jiang R., Jin S., Geng Z. (2017). Resveratrol alleviates heat stress-induced impairment of intestinal morphology, microflora, and barrier integrity in broilers. Poult. Sci..

[57] Lv T., Shen L., Yang L., Diao W., Yang Z., Zhang Y., Yu S., Li Y. (2018). Polydatin ameliorates dextran sulfate sodium-induced colitis by decreasing oxidative stress and apoptosis partially via Sonic hedgehog signaling pathway. Int. Immunopharmacol..

[58] Zeng Z., Yang Y., Dai X., Xu S., Li T., Zhang Q., Zhao K.-S., Chen Z. (2016). Polydatin ameliorates injury to the small intestine induced by hemorrhagic shock via SIRT3 activation-mediated mitochondrial protection. Expert Opin. Ther. Targets.

[59] Bereswill S., Muñoz M., Fischer A., Plickert R., Haag L.-M., Otto B., Kühl A.A., Loddenkemper C., Göbel U.B., Heimesaat M.M. (2010). Anti-inflammatory effects of resveratrol, curcumin and simvastatin in acute small intestinal inflammation. PLoS ONE.

[60] Ling K.-H., Wan M.L.Y., El-Nezami H., Wang M. (2016). Protective capacity of resveratrol, a natural polyphenolic compound, against deoxynivalenol-induced intestinal barrier dysfunction and bacterial translocation. Chem. Res. Toxicol..

[61] Caruso R., Marafini I., Franzè E., Stolfi C., Zorzi F., Monteleone I., Caprioli F., Colantoni A., Sarra M., Sedda S. (2014). Defective expression of SIRT1 contributes to sustain inflammatory pathways in the gut. Mucosal Immunol..

[62] Wellman A.S., Metukuri M.R., Kazgan N., Xu X., Xu Q., Ren N.S., Czopik A., Shanahan M.T., Kang A., Chen W. (2017). Intestinal epithelial sirtuin 1 regulates intestinal inflammation during aging in mice by altering the intestinal microbiota. Gastroenterology.

[63] Singh U.P., Singh N.P., Singh B., Hofseth L.J., Price R.L., Nagarkatti M., Nagarkatti P.S. (2010). Resveratrol (trans-3, 5, 4′-trihydroxystilbene) induces silent mating type information regulation-1 and down-regulates nuclear transcription factor-κB activation to abrogate dextran sulfate sodium-induced colitis. J. Pharmacol. Exp. Ther..

